# Mobile Health in Urology: The Good, the Bad and the Ugly

**DOI:** 10.3390/jcm9041016

**Published:** 2020-04-03

**Authors:** Nuno Pereira Azevedo, Stavros Gravas, Jean de la Rosette

**Affiliations:** 1 Department of Urology, Entre o Douro e Vouga Medical Center, 4520-211 Santa Maria da Feira, Portugal; 2 Department of Urology, Faculty of Medicine, School of Health Sciences, University of Thessaly, 411-10 Larissa, Greece; sgravas2002@yahoo.com; 3 Department of Urology, School of Medicine; Istanbul Medipol University, TEM Avrupa Otoyolu Göztepe Çıkışı No: 1 Bağcılar, 34214 Istanbul, Turkey; j.j.delarosette@gmail.com

**Keywords:** urology, mHealth, eHealth, apps, applications

## Abstract

Our aim is to present the current position of mobile health (mHealth) and the delivery of healthcare services via mobile communication devices in urology. We conducted a literature review of urology mHealth papers on PubMed. Results indicate that mHealth is becoming ubiquitous in contemporary healthcare systems. Although its potential has been shown, urology lags behind other areas, representing just 0.1% of the 300,000 available medical apps in the Apple App Store and Google Play Store. Furthermore, there is a lack of expert healthcare professional involvement in app development. To avoid harm, it is critical that the scientific accuracy, patient privacy, and user safety of urology mHealth applications are assured. This is because there is no globally enforced medical app regulation, compulsory scientific guidelines, nor mandatory industry standards. Urologists, either individually or via scientific organizations, should have a pivotal position in the design, development, review, certification, and recommendation of apps. mHealth holds great potential in urology, as it can aid multiple stakeholders: citizens, patients, healthcare professionals, health organizations, and public authorities (e.g., Ministry of Health). Even though it is mostly used to improve existing medical activities at present, the future will include revolutionary and ground-breaking technology solutions. This innovative field should be seen by urologists as an opportunity to provide greater care to our patients and better tools and knowledge to our peers.

## 1. Introduction

Information and communication technologies (ICT) can offer patients and healthcare providers new ways to improve wellness, practice prevention, and reduce suffering from diseases. The World Health Organization (WHO) defines eHealth as “the use of information and communication technologies (ICT) for health” [1]. Mobile health (mHealth), which is a subset of eHealth, is described by the WHO Global Observatory for eHealth “as medical and public health practice supported by mobile devices, such as mobile phones, patient monitoring devices, and other wireless devices” [2]. Because of its ease of use and extensive acceptance, mHealth is considered to be a valuable tool in the implementation of patient-centered care (patient-reported preferences, experiences, and outcomes), which has become a goal of contemporary healthcare systems and international standards [3].

Since January of 2007, when Apple’s founder Steve Jobs unveiled the iPhone with an enticing preamble: “Every once in a while, a revolutionary product comes along that changes everything”, both Apple (Cupertino, California, United States) and Google (Mountain View, California, United States)—which together represent more than 90% of the smartphone market—supported specific development environments for their operating systems. Since both Apple and Google promoted mHealth as a top-priority, a plethora of mHealth apps became available for both healthcare professionals and lay people [4,5,6].

A seminal review of urology apps available in the Apple App Store and Google Play Store that was performed in 2015 identified 150 urology apps, representing just 0.15% of the total number of smartphone medical apps available at the time (approximately 100,000) [7]. In December 2017, after a 200% growth in the number of mHealth apps, an updated urology review was performed, which identified a 17% increase in the number of urology apps: 67 (38%) available exclusively for Android, 62 (35%) only for iOS, and 47 (27%) presented on both stores (Table 1) [8]. However, there are currently over 300,000 mHealth apps available in the Apple App Store and Google Play Store, which shows there is a significant discrepancy between the increase in all mHealth and urology apps, that is, 200% and 17%, respectively [9].

Given the rapid implementation of mHealth and the many opportunities for its use in the medical field, this development deserves a SWOT analysis (a strategic planning technique used to identify strengths, weaknesses, opportunities and threats), followed by a concise discussion.

### 1.1. Strength of mHealth

One of the biggest strengths of mHealth is that it envisions healthcare delivery that overcomes geographical, temporal, and organizational barriers. mHealth is based on easily scalable solutions (e.g., online platforms) accessed using readily available equipment (e.g., smartphones), which has the potential to decrease healthcare costs. Therefore, it can promote socio-economic inclusion and equality, quality of life and patient empowerment through greater transparency, and access to services and information. In addition, mHealth can facilitate other ways of delivering care, such as virtual consultations to replace in-person visits (i.e., telemedicine), which is a viable solution in several contexts, such as during disasters and pandemics (e.g., COVID-19).

mHealth has a broad range of presentations, and successful evidence-based mHealth interventions include medication adherence (e.g., improving the cardiometabolic profile of patients with hypertension) and promoting smoking cessation (e.g., via text messaging) [10,11]. Moreover, there is evidence of successful implementations of mHealth in low-resource contexts, such as mobile-phone-based clinical guidance for rural health providers in developing countries [12]. This is possible because mHealth’s dynamic infrastructure can easily overcome geographical barriers and environmental circumstances: currently there are places where people are more likely to have access to a smartphone than to clean water or electricity [13]. Furthermore, mHealth’s demographic reach transcends generations, as demonstrated by various successful examples, including the promotion of physical activity and its acceptance by both young and older adults [14,15].

### 1.2. Weakness

Few mHealth apps have been scientifically reviewed and/or approved by the European Medicines Agency or the USA Food and Drug Administration, as most mHealth apps are not considered medical devices by their developers [16]. Subsequently, they bypass strict regulation, such as the European Union “Guidelines on the qualification and classification of standalone software used in healthcare within the regulatory framework of medical devices”, which states: “it is necessary to clarify that software in its own right, when specifically intended by the manufacturer to be used for one or more of the medical purposes set out in the definition of a medical device, is a medical device”.

At present, a cause for concern is that even when there is reference to a healthcare professional or a urological society’s involvement in the app design, it is not possible to validate if this is true or not, nor to systematically assess their level of responsibility as either an external advisor, major stakeholder, or sole author. Additionally, there are no available tools or evidence on how to quantify this information in a reproducible method.

While mHealth apps are becoming increasingly popular for both professionals and patients, concerns have been raised about apps’ scientific accuracy and user security, which are exacerbated by the lack of regulation. Several pitfalls have been identified, such as apps crammed with outdated (mis)information created by lay people, with disregard for usability and scientific evidence; there is also the risk of possible charlatans [7]. Actual examples have been identified that can be dangerous to uninformed users, either by inaccuracy (e.g., miscalculation when using an opioid dose calculator) or by making false claims, such as dermatology apps that claim to diagnose skin cancer in spite of evidence that they misclassify 80% of textbook melanomas and apps that guarantee they can cure breast cancer [17]. Although these concerns have attracted the attention of public entities such as the European Union and the US Food and Drug Administration, there is still no mandatory certification for mHealth apps.

### 1.3. Opportunities

mHealth can also be used for conducting research, educating professionals, and monitoring public health. Both healthcare professionals and developers should strive to future-proof their designs by enabling their mHealth projects to incorporate new practices and pioneering research, therefore becoming resilient via innovation. In the near future, we expect eHealth and mHealth to become intrinsic to healthcare as a whole. For example, real-time monitoring devices can gather live data from sensors and send inputs into a mobile medical app on a smartphone, server, or network to support clinical decision making [7]. This new wave of big data powering personalized risk assessment tools will help patients and healthcare professionals make enhanced shared decisions. However, to avoid harm, it is critical that, among other concerns, the scientific accuracy, patient privacy, and user safety of mHealth applications are assured. Moreover, both lay and professional users should be aware that mHealth tools are designed to support and enhance, but will probably never fully replace, a direct consultation with a healthcare professional.

### 1.4. Threats

Privacy, security, and reliability are of paramount importance, as there is no global standard medical app regulation, nor are there compulsory scientific guidelines or mandatory industry standards. There is even a substantial contrast between Google’s app review process (i.e., Android apps are automatically accepted) and Apple’s (i.e., iOS apps are only made available after technical approval by Apple staff). For example, iOS apps that try to access personal data (e.g., phone calls, SMS, or location services) without user consent are rejected [4].

## 2. Discussion

Contemporary literature has shown a lack of involvement of healthcare professionals in app development in several medical specialties, including urology, even though it has also been proven that their participation increases content accuracy, app downloads, and buy-in [7,18]. With the growing number of available apps, the challenge is finding safe and well-designed apps. Another concern is the involvement of commercial companies in this type of media, with worries about their funding and purpose [19]. A particular apprehension is the potential bias in product promotion, which can ultimately create conflicts of interest between the developer, healthcare professionals, and the end user.

To address these problems, it has been suggested that healthcare professionals should have a pivotal position in the design, development, review, certification, and recommendation of mHealth apps [20]. This can either be done individually or through scientific societies, which could coordinate this effort. A pragmatic stand has been taken by the American Psychiatric Association (APA), which developed a step-by-step App Evaluation Model [21], in which psychiatrists are advised to:

(1) Begin by collecting background information on the app (e.g., who is the developer, what is the business model);
(2) Exclude risk, privacy, and security issues (e.g., does the app have a privacy policy, which personal data is collected, is data available to any third party?);
(3) Evaluate the evidence (e.g., is there peer-reviewed, published evidence about the app or the science behind it?);
(4) How easy is it to use? (i.e., evaluate its usability);
(5) Assess interoperability (i.e., how easy is it to share the data in the app with other healthcare software?).

App content analysis (i.e., comparing an app’s information and features with clinical guidelines and evidence-based protocols) should be performed on every mHealth app before they are made available to the general public, even if the app was developed with contributions from healthcare professionals. However, most mHealth apps are not developed by large enterprises: one-third are developed by single individuals, and another third by small companies (i.e., with staff between two and nine employees) [22]. Some developers might not have the resources to include a healthcare professional in the app’s team. Developing a scientifically valid and appropriately designed mHealth app might require input from experts in several fields of science, and healthcare professionals have the deontological duty to remain stakeholders in this process. The level of scrutiny should match the potential health hazards, and the degree of regulation should be proportional to the clinical implication derived from the app, ranging from low (e.g., apps that give access to online medical journals, which only show content that has already been peer-reviewed) to high (e.g., apps that dispense clinical advice).

mHealth in urology is still in its infancy, like in many other medical disciplines. Currently, this new technology is mostly used to improve and optimize existing medical activities, but one of the most promising areas where mHealth in urology could be developed is patient self-management. Implementing a shared-decision model process changes the quality of patient–provider communication and can improve patients’ satisfaction not only with the health decision per se, but also with the process itself [23]. Studies have shown that cancer patients who are more engaged in their health decisions are more likely to be satisfied with their choices [23].

Even though engaging patients is positively associated with better quality of life outcomes, in addition to being considered ethically important and actively promoted by health policies, as well as endorsed by scientific guidelines, in clinical practice, the implementation of shared decision making has historically been infrequent [24]. Currently, there is a tendency to endorse shared decision making in medicine, including in urology (e.g., prostate-specific antigen (PSA) testing).

Moreover, there is a need to make the most of health information technology, and urology can be at the forefront of this effort—namely, with the development of decision aids, which can be used in the process of shared decision making, to improve patients’ knowledge regarding options, promote a more active role in decision making, and increase their risk perception [25]. The self-management of health issues by citizens is contingent on public awareness, as well as on the information made available to lay people.

In a contemporary systematic review of the factors that determine the success and failure of eHealth interventions, a causality between design and outcome was demonstrated [26]. Moreover, the involvement of users (i.e., user-centered design)—either healthcare professionals or lay people—during the design was central to the eHealth intervention’s success [26]. We can presume that the same is true for mHealth.

Patients feel empowered when they take a more active part in their healthcare decisions, which in turn increases their adherence to care plans, improves clinical outcomes, and reduces healthcare costs (e.g., a successful eHealth implementation improved the glycemic control of diabetic patients) [27,28]. Patients also prefer decision support tools over usual care, as it improves their confidence regarding available options, while reducing their knowledge gap [25]. As such, mHealth is fitting for the current model of patient-centered care: the future of patient empowerment will be facilitated by technological advancements and better access of patients to these technologies [29].

Recently, Apple unveiled a new plan with the title “Empower your patients with Health Records on iPhone, asking organizations to allow patients to download their medical data into their smartphones [30]. Coincidently, a few days later, Amazon (Seattle, Washington, United States), Berkshire Hathaway (Omaha, Nebraska, United States), and JPMorgan Chase & Co. (New York, New York, United States) announced a non-profit business partnership dedicated to improving their U.S. employees’ wellbeing and healthcare satisfaction, while reducing overall costs [31]. Warren Buffett, Berkshire Hathaway Chairman and Chief Executive Officer, summed up their impetus to act: “The ballooning costs of healthcare act as a hungry tapeworm on the American economy” [31]. These two initiatives are a clear statement that future healthcare will have a strong technological tonic. In the case of mHealth, it is expected that we will move beyond the current separate insulated platforms and single apps, and into a true health ecosystem [32]. We praise this change in paradigm in global healthcare and expect a parallel shift in urology.

mHealth will likely help in the transition from isolated healthcare sources with inaccessible proprietary information silos to an integrated paradigm of continuous care and symbiotic services built around the individual, with an improvement in health outcomes and reduction in costs. However, for that to happen, cooperation is vital: in the past, research was based on clinical data that was mostly produced by the healthcare professional; currently, we have access to vast amounts of patient-produced data, not only from apps, but also from wearable devices and online platforms. It is essential to capitalize on all this new information and use it to provide better personalized care.

Until official certification is enforced for mHealth apps, urological societies could take part in the regulatory process by publishing mHealth recommendations, like what was done for social media [19,33]. Healthcare expert involvement in app development could be analogous to a “quality mark”, assuring that the app is safe and scientifically valid. However, until there is a secure way to verify the authenticity of these claims, unscrupulous app developers could potentially exploit this by deceptive advertising or false endorsement (e.g., hiring a healthcare professional just to mention it, even though the app may not be related to their field of expertise). The lack of a standardized format for the disclosure of expert participation and the absence of readily available tools to verify and quantify it should be addressed. Alternatively, Apple and Google could require mHealth apps to be reviewed and/or approved by the European Medicines Agency, the United States of America Food and Drug Administration, or, if applicable, the Health Insurance Portability and Accountability Act (HIPAA) regulation. Moreover, urology societies could issue an official scientific seal or maintain a register of board-certified apps.

A good example of an mHealth app designed by urologists is the MyBPH Care app. The app, available for Apple iOS and Google Android platforms, is being evaluated by uCare, the Research Office of the Société Internationale d’Urologie (SIU), as part of a clinical trial (ClinicalTrials.gov ID: NCT03228485), to assess the feasibility and acceptability of a mobile application for men with lower urinary tract symptoms and/or benign prostate hyperplasia (LUTS/BPH). It was developed to facilitate and improve the management of patients with LUTS/BPH, to increase medication adherence, and to improve communication between patients and physicians.

To achieve this, MyBPH Care includes features such as a medication reminder (i.e., the patient receives a notification to take their medication daily), a messenger function (allowing instant communication between patient and physician), and patient-reported outcome monitoring via questionnaires such as the International Prostate Symptom Score (IPSS), the International Index of Erectile Function (IIEF), and the Short Form 36 (SF-36, a general questionnaire about quality of life).

MyBPH Care is expected to be useful in daily clinical practice and, in addition to all scientific and ethical safeguards, the app is compliant with the European Commission’s General Data Protection Regulations (GDPR) laws and the United States’ HIPAA Act. Data safety is guaranteed, as the app uses a safe, validated, secured server to store medical data (ISO 27001 compliant). Moreover, it provides a concrete example of the benefits of having urologists involved in the design and development of mHealth apps. To promote the uptake of mHealth apps, it is important to involve professionals in their design and development, to assure their usefulness and usability, and also to guarantee the safety and privacy of their users and their data.

Apps represent an innovative opportunity to enhance patient care in urology. Because it is impossible to verify all available apps, maintaining a peer-reviewed register of certified urology apps or issuing an official seal of approval could influence overall app design and, consequently, improve mHealth in urology.

## 3. Conclusions

mHealth holds great potential as it can aid multiple stakeholders: citizens, patients, healthcare professionals, health organizations, and public authorities. Future developments will include the use of innovative and ground-breaking ICT solutions. However, to increase the uptake of mHealth, it is important that healthcare professionals are involved in their design, assuring usability, and also their development, promoting evidence-based views. It is critical that accuracy, privacy, and safety are assured. mHealth app development should be seen by urologists as an opportunity to provide greater care to our patients and better tools and knowledge to our peers.

## Figures and Tables

**Table 1:** Comparison of available apps between January 2015 and December 2017.

	January 2015	December 2017	Change over Time
Total Number of Apps	100,000	300,000	(+200%)
Urology Apps	150	176	(+17%)
Apple	44 (29%)	62 (35%)	(+21%)
Android	56 (37%)	67 (38%)	(+3%)
Both	50 (33%)	47 (27%)	(−18%)
			
Healthcare Participation	119 (80%)	132 (75%)	(−6%)
